# Oregano and Thyme Essential Oils Encapsulated in Chitosan Nanoparticles as Effective Antimicrobial Agents against Foodborne Pathogens †

**DOI:** 10.3390/molecules26134055

**Published:** 2021-07-02

**Authors:** Giuseppe Granata, Stefano Stracquadanio, Marco Leonardi, Edoardo Napoli, Graziella Malandrino, Viviana Cafiso, Stefania Stefani, Corrada Geraci

**Affiliations:** 1 Istituto di Chimica Biomolecolare-C.N.R., Via Paolo Gaifami 18, 95126 Catania, Italy; giuseppe.granata@icb.cnr.it (G.G.); marco.leonardi@phd.unict.it (M.L.); edoardo.napoli@icb.cnr.it (E.N.); 2 Department of Biomedical and Biotechnological Sciences, University of Catania, Via S. Sofia 97, 95123 Catania, Italy; s.stracquadanio@hotmail.it (S.S.); vcafiso@unict.it (V.C.); stefania.stefani@unict.it (S.S.); 3 Department of Chemistry, University off Catania, Via A. Doria 6, 95125 Catania, Italy; gmalandrino@unict.it

**Keywords:** natural food preservatives, chitosan nanoparticles, essential oils, antibacterial activity, ecofriendly nanosystems

## Abstract

The use of natural compounds with biocidal activity to fight the growth of bacteria responsible for foodborne illness is one of the main research challenges in the food sector. This study reports the preparation and physicochemical characterization of chitosan nanoparticles loaded with *Thymus capitatus* (Th-CNPs) and *Origanum vulgare* (Or-CNPs) essential oils. The nanosystems were obtained by ionotropic gelation technique with high encapsulation efficiency (80–83%) and loading capacity (26–27%). Nanoparticles showed a spherical shape, bimodal particle size distribution, and good stability (zeta potential values > 40 mV). The treatment of the nanosuspensions at different temperatures (4 and 40 °C) and storage times (7, 15, 21, and 30 days) did not affect their physicochemical parameters and highlights their reservoir ability for essential oils also under stressful conditions. Both Or-CNPs and Th-CNPs exhibited an enhanced bactericidal activity against foodborne pathogens (*S. aureus*, *E. coli*, *L. monocytogenes*) than pure essential oils. These ecofriendly nanosystems could represent a valid alternative to synthetic preservatives and be of interest for health and food safety.

## 1. Introduction

The lifestyle of the most industrialized countries leads people to increase their consumption of meals outside their homes (ready-to-eat foods). At the same time, consumers are strongly oriented towards the consumption of food with characteristics of safety and quality. The globalization of the food supply chain has led to the spread of foodborne pathogens in different areas of the world. Furthermore, the use of synthetic antiseptics and germicides in food processing can favor the development of microorganisms resistant to the traditional treatments.

In this context, foodborne diseases that involve larger and larger segments of the population, especially among the elderly and people with immune system deficiencies, are a global public health challenge. This problem is very closely related to the practices in the food industries. In fact, these companies are strongly motivated to find more sustainable alternative solutions to synthetic preservatives, and to ensure the safety of the product distributed on the market.

Outbreaks of foodborne diseases are caused by different pathogens, such as *Staphylococcus aureus*, *Escherichia coli*, and *Lysteria monocytogenes*, which can produce toxins in foodstuff and after ingestion in gastro-intestinal compartment, causing various diseases, including toxic shock syndrome, endocarditis, meningitis, encephalitis, cholecystitis, septicemia, and urinary tract infection [1,2].

The use of natural compounds with biocidal activity to fight the growth of bacteria responsible for foodborne illness is one of the main challenges that research in food sector addresses. Essential oils (EOs), containing natural bioactive substances, are widely studied for their functional characteristics and therapeutic effects. EOs have commercial importance for the industries operating in cosmetics, perfume, food, sanitary, agronomic, and pharmaceutical sector [3].

They are obtained by the extraction of different aromatic plants, such as spices and herbs, and contain about 20 to up to more than 100 constituents at very different concentrations depending on many factors, including harvest season, soil composition, climate, and the technique of oil extraction. EOs have well-known antimicrobial properties, mainly attributable to major components, including terpenoids, phenylpropanoids, and short chain hydrocarbon derivatives at low molecular weight. Some examples about their use as a food preservative to reduce pathogenic bacteria and to produce healthy food have been reported in the literature [4,5].

Due to the multicomponent nature of EOs, their antimicrobial activity is not attributable to a specific mechanism; instead, it is the result of the action on multiple targets in the cells. For this reason, they do not cause bacterial resistance and are suitable to fight multi-drug resistant bacteria [6,7,8].

The EOs, due to their hydrophobicity, are generally able to disrupt the structure of the bacterial cell membranes, making them more permeable, causing leakage of cytoplasmic constituents such as electrolytes and metabolites. Moreover, EOs can act on the enzymes involved in the energy regulation or synthesis of structural components [9].

Therefore, the use of EOs to contrast the development of fastidious microorganism could represent a valid alternative to use of chemical preservatives in food and to prepare food free from synthetic additives.

Few food preservatives based on EOs are already on the market [4], but sometimes, the exact composition of bioactive components is not known [10]. Furthermore, an EO quantity greater than that required for the in vitro antimicrobial test must be added to the food to obtain an effective preservative activity [11]. This phenomenon could be explained by the presence of high levels of fat and/or protein in food, which make EOs less available in the aqueous phase, where bacteria are mostly located. Moreover, the physical structure of food (i.e., gel matrix) could influence the antimicrobials capability of EOs to interact with microbial cells [12,13].

Nanoencapsulation of EOs represents a valid and efficient strategy to circumvent these obstacles, improving their bioaccessibility and bioavailability, protecting them from degradation phenomena (light, oxygen, temperature and pH), increasing solubility and physical stability, decreasing volatility, and masking the intense aroma. Differently to large capsules, the nanocapsules have a subcellular size, a larger surface area per unit volume, and potential enhancement of EO concentration in food areas where the microorganisms are preferentially located, such as water-rich phases or liquid-solid interfaces [14].

Given the great interest in biopolymers, we have focused our attention on chitosan polysaccharide to prepare new stable nanoparticles as a reservoir of essential oil with antimicrobial activity for a prolonged release over time, in order to promote their applications in the health and food safety sector.

Chitosan, generally recognized as safe (GRAS) and approved as a food additive by the Food and Drug Administration [15], possesses a polymeric structure composed of randomly distributed units of D-glucosamine and *N*-acetyl-d-glucosamine linked *via* β (1–4) glycosidic bond. It is an inexpensive cationic biopolymer obtained by deacetylation of chitin, a polysaccharide found in many kinds of crustacean shells as well as in the cell walls of fungi. Chitosan has several advantages, including nontoxicity, mucoadhesivity, exceptional biocompatibility and biodegradability, antibacterial activity, and antioxidant effects [16]. These peculiarities make a chitosan-based carrier suitable for use in medicine but also in food preparation [17]. Some examples of EO-loaded chitosan nanoparticles with potential applications as natural food preservatives are reported in the literature. Hu et al. [18] prepared chitosan nanoparticles loaded with cinnamon EO capable to exhibit excellent antimicrobial and antioxidant property during food-chilled storage. Other authors reported that chitosan nanoparticles incorporating *Citrus aurantium* EO preserved the food postharvest quality [19]. Sotelo-Boyás et al. incorporated lime EO in chitosan nanoparticles and chitosan nanocapsule for potential use in coatings or packaging to prevent foodborne pathogen contamination in food [20]. The improvement of preservation of fruit in term of fungal decay by using chitosan-essential oils film or nanoemulsions was described by Grande-Tovar et al. [21]. Barrera-Ruiz et al. [22] reported chitosan nanoparticles loaded with cinnamon, thyme, and *Schinus molle* essential oils that were effective against some foodborne pathogens. Chitosan nanoparticles containing thyme essential oil were prepared by Barzegar et al. [23]. These nanocapsules showed enhancement of the thermal stability and antioxidant activity of EO. Hadidi et al. synthesized clove essential oil encapsulated into chitosan nanoparticles, showing greater antimicrobial and antioxidant activity than free EO, for active food packaging [24].

Among the methods to prepare chitosan nanoparticle the ionotropic gelation is the most widely used due to the mild processing condition and the aqueous environment. In this technique, a polyanion is used to interact, by electrostatic interaction, with the positively charged primary amino groups on chitosan to provide spherical chitosan nanoparticles (CNPs).

Here, we reported the capability of chitosan nanoparticle, prepared by the ionotropic gelation technique using sodium tripolyphosphate (TPP), to encapsulate the essential oil from *Thymus capitatus* (Th) and *Origanum vulgare* (Or), which are generally recognized as safe (GRAS) compounds by Food Drug Administration [25]. The nanostructured systems were characterized for morphology, particle size, zeta potential, loaded amount of bioactive, encapsulation efficiency, and stability over time. The antimicrobial activity against some pathogens widely spread in different environments, and was also evaluated in comparison with pure essential oils in order to achieve potential natural preservative nanosystems that may find practical applications in the food sector.

## 2. Results and Discussion

### 2.1. Determination of the Degree of Deacetylation (DD) of Chitosan

The DD of chitosan is a very important parameter, which affects the physicochemical characteristics and the biological properties of the chitosan [26]. Moreover, it is crucial in addressing the specific applications of the polymer. It is known that the presence of a high number of primary NH_2_ groups compared to the *N*-acetylglucosamine moieties improves the biological properties of the polymer [27]. Sometimes, the DD values of chitosan, declared by the commercial supplier, are in a fairly wide range, and it is good practice to check this first. Proton nuclear magnetic resonance (^1^H-NMR) spectroscopy is one of the techniques used to determine the DD of chitosan. This technique has been selected by the American Standard Test Method organization (ASTM, edition 2003, test F2260-03) as a standard method for its high sensitivity and precision [28].

^1^H-NMR spectra allow to obtain the DD percentage values of the polymer by the integrations of proton signals, belonging to the acetylated and deacetylated units forming the polymer. As reported in Figure S1, the chitosan used in this work has DD value of 76% ± 1, calculated using Equation (1) (see Section 3.2).

### 2.2. Volatile Composition of Origanum vulgare ssp. hirtum and Thymus capitatus Oils

The essential oils from *Origanum vulgare ssp. hirtum* and *Thymus capitatus*, two very typical Sicilian aromatic plants, were selected for their different chemotypes. In particular, oregano essential oil (Or-EO) was a thymol chemotype, while thyme essential oil (Th-EO) was a carvacrol chemotype. The chemical composition of these EOs is described in our previous work regarding their nanoencapsulation in a different polymer [29] and is showed in Table S1. Useful details for the discussion are described below.

In the Or-EO, the most represented chemical class are oxygenated monoterpenes (52.1%), mainly due to the high presence of thymol (more than 43%). In addition, it contains high quantities of the monoterpene hydrocarbons *p*-cymene (14.4%) and *γ*-terpinene (15.4%). These two compounds (which are thymol’s biosynthetic precursors [30]), contribute to the high percentages of the total monoterpene hydrocarbons (40.3%). Sesquiterpenes is the least represented class, with a total of 7.4% as the sum of at least 16 compounds, all below 2%. This chemical profile is in accordance with typical Sicilian wild oregano essential oil composition [31,32].

The Th-EO has a chemical composition quite different from that of oregano. The most represented chemical class still consists of oxygenated monoterpenes with a higher percentage (76.6%), which is almost totally dominated by the presence of carvacrol (73.0%). A negligible percentage of thymol is present (0.3%). This is reflected in the quantities of the total hydrocarbon monoterpenes, which drop to 14.7%. Similarly, the percentages of *p*-cymene and γ-terpinene drop, respectively, to 6.9% and 2.5%.

The amount of total sesquiterpenes is comparable between the two essential oils. The only difference is that in the thyme EO, this class is composed almost exclusively of β-caryophyllene, which reaches 5.5%, and at least 11 other components, all below 1%. This composition is in accordance with what has already been widely reported for Sicilian wild thyme essential oils [33].

### 2.3. Preparation and Physicochemical Characterization of EO-CNPs

Chitosan nanoparticles were obtained at room temperature by ionic gelation technique, using tripolyphoshate (TPP) [34]. This polyanion was selected for the quick gelling capability at room temperature and non-toxic property [35,36]. In the formulation of EO-CNPs, polysorbate 80 was used to emulsify the essential oil (oregano or thyme) in chitosan solution. The non-ionic surfactant is able to reduce the surface tension between the two phases, and prevent the nanoparticle aggregation by a steric effect. During the cross-linking process, droplets of dispersed oil are captured and incorporated between the polymer meshes, thus forming a milky suspension containing EO-CNPs.

**Figure 1:**
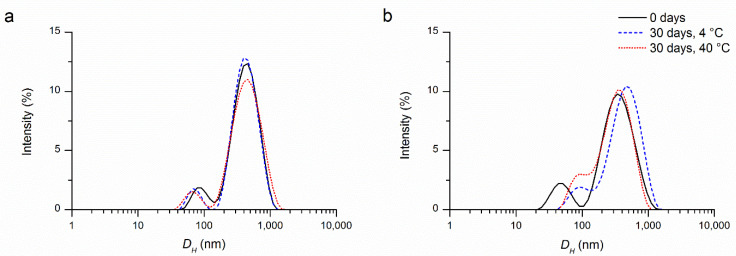
Intensity weighted distribution of the hydrodynamic diameter (*D_H_*) of (**a**) Th-CNPs and (**b**) Or-CNPs at different storage conditions.

The distribution of the hydrodynamic diameter (*D_H_*) of Th-NCPs and Or-NCPs, obtained by DLS experiments (Figure 1), was indicative of a bimodal size distribution for both nanosystems, with peaks at 86 nm and 449 nm for Th-NCPs, and 62 nm and 407 nm for Or-NCPs.

As reported for other chitosan nanoparticles, the observed PDI values lower than 0.7 (0.42 for Th-NCs and 0.53 for Or-NCs) confirm the good acceptability of the analyses performed and the homogeneity of the sample [37,38].

The Z potential, related to the charges available on the nanoparticle surface, also provides useful information on the stability. The value is obtained by electrophoretic mobility measurement of the nanoparticles that move under the influence of an electric field. For Th-CNPs and Or-CNPs nanosystems, Z potential values of +44 ± 2 mV and +46 ± 2 mV were observed, respectively (Tables S2–S5), and no phenomenon of aggregation and flocculation was detected in both nanoparticle suspensions. It is known that a high zeta potential greater than 30 mV (absolute value) [39] makes the nanoparticles repel each other so as to ensure physical colloidal stability of the suspensions. Moreover, the positively charged chitosan nanoparticles, due to the protonation of NH_2_ groups (NH_3_^+^ groups) under acidic conditions, is an important factor for their antibacterial activity [40].

The rheological properties of nanoparticles with food applications play an interesting rule in the manufacturing and quality control of food products [41]. Rheology experiments carried out on EO-CNP suspensions showed that their apparent viscosity decreased with increasing the shear rate at low shear rate values suggesting non-Newtonian properties for both suspensions, while at high shear rate the viscosity values became constant (Newtonian behavior), reaching 1.6 mPa s and 1.4 mPa s for Th-CNP and Or-CNP suspensions, respectively (Figure S2).

**Figure 2:**
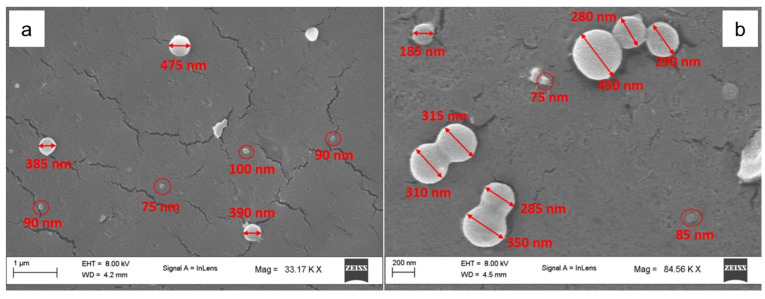
SEM image of (**a**) Or-NCPs and (**b**) Th-NCPs.

The morphology of chitosan nanoparticles has been probed through field emission scanning electron microscopy (FE-SEM). The FE-SEM images of the Or-NCPs (Figure 2a) and Th-NCPs (Figure 2b) showed spherical nanoparticles and a bimodal size distribution (large and small nanoparticles). The average size values of nanoparticles agree quite well with those obtained by DLS measurements.

#### 2.3.1. Encapsulation Efficiency (EE%) and Loading Capacity (LC%)

The EE represents an important parameter that characterizes the quality of nanocapsules. The EE*%* of Th-CNPs and Or-CNPs were 80 ± 2% and 83 ± 3%, respectively. These data reveal a good encapsulation efficiency for both the EOs. In this regard, Sotelo-Boyás et al. [42] reported a very similar value (80.5 ± 1%) for Th-CNPs, whereas the *EE*% value obtained by us for Or-CNPs is much higher than described by other authors. For example, Hosseini et al. reported that the *EE%* of oregano essential oil in chitosan nanoparticle can range from 5.45% to 24.72% [43].

Good loading capacity values were also found for both nanoparticle suspensions, specifically 26 ± 1% for Th-CNP and 27 ± 1% for Or-CNPs.

#### 2.3.2. Physicochemical Stability of EO-CNPs

**Figure 3:**
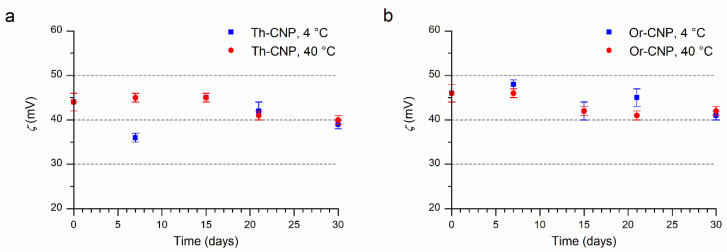
Zeta potential over time at 4 °C (square) and 40 °C (circle) of (**a**) Th-CNPs, (**b**) Or-CNPs.

To evaluate the physicochemical stability of Th-CNPs and Or-CNPs suspensions over time and storage, parameters such as hydrodynamic diameter, zeta potential, and retention percentage of encapsulated EOs were determined at 7, 15, 21, and 30 days of storage and temperatures of 4 °C and 40 °C, according to the methods reported by us in previous works [29,44]. In particular, for both nanosuspensions, it was observed that the particle bimodal distribution and size of the two populations (small and large particles) did not change significantly, even when the time and temperature of storage were varied. Similarly, the zeta potential remains at high positive values (around 40 mV), indicating no tendency to aggregation for both nanosuspensions (Figure 3). These results suggest that both nanosystems are stable at 4 °C and 40 °C for a storage period of 30 days (Tables S2–S5).

**Figure 4:**
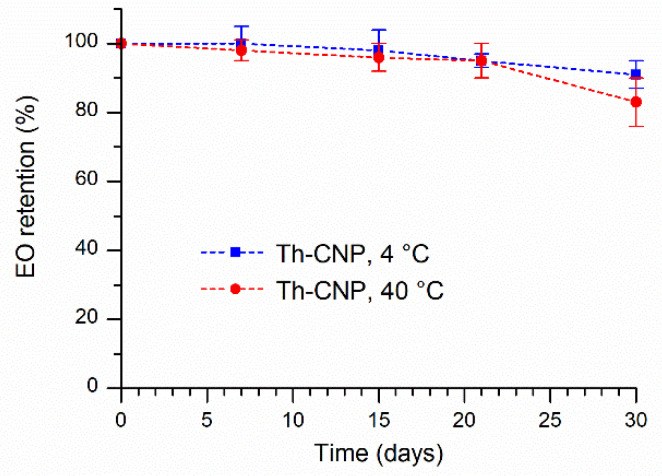
Essential oil retention over time at 4 °C (square) and 40 °C (circle) for Th-CNPs.

Regarding the retention of thymus and oregano essential oil from the chitosan-based nanoparticles, the different behavior of the two systems was highlighted. For Th-CNPs, as shown in Figure 4, the retention decrease of thyme essential oil occurs gradually up to 21 days for both samples kept at 4 °C and 40 °C, reaching the 95% retention value. At 30 days, an EO retention of 91% and 83% is observed for samples kept at 4 °C and 40 °C, respectively.

**Figure 5:**
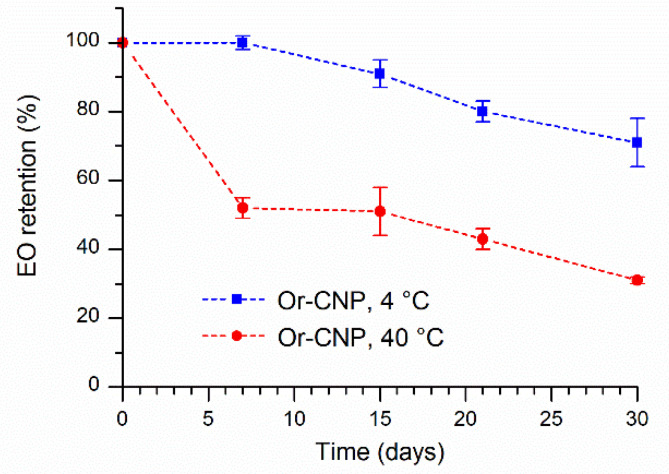
Essential oil retention over time at 4 °C (square) and 40 °C (circle) for Or-CNPs.

Unexpectedly, Or-CNPs shows a recognizably different phenomenon. After 7 days of storage at 4 °C temperature, the EO content in nanoparticles was almost unchanged, while the Or-CNPs sample kept at 40 °C showed only 52% of EO retention. Successively, decreasing trend in the content of oregano essential oil into nanoparticles was observed, reaching, at 30 days, retention values of 71% and 31% at 4 °C and 40 °C, respectively (Figure 5).

Therefore, the Th-CNP system which shows a better controlled and prolonged release of EO is more stable and robust than the one containing oregano essential oil. The trend observed for Or-CNPs was unexpected when compared with the results reported in our previous work in which the essential oils of thyme and oregano are confined within the core of polycaprolactone-based nanocapsules [29]. This could be attributable to the chitosan nanocarrier system that incorporates essential oil between the polymer meshes. Probably, at a stress temperature (40 °C), the different chemical composition and physical properties (e.g., volatility) of EO could make a difference. More in-depth studies are needed to understand this behavior.

### 2.4. Antimicrobial Activity of Th-CNPs and Or-CNPs

The in vitro antimicrobial activity of pure EOs *Thymus capitatus* and *Origanum vulgare*, Th-NC and Or-NC suspensions, and unloaded chitosan nanoparticles (CNPs) was evaluated against three selected food poisoning pathogens, i.e., *S. aureus*, *L. monocytogenes*, and *E. coli*, by MIC and MBC assays. The use of both these assays allows us to know if the activity observed is bacteriostatic and/or bactericidal. This is particularly important for the application in the food industrial sector (packaging, processing, safety, etc.).

As reported in Table 1, both chitosan nanoparticles loading essential oils showed a strong increase in antibacterial activity compared to pure essential oils. For Th-CNPs, the lowest MIC value (0.03 mg/mL) was obtained for *L. monocytogenes*, while for *S. aureus* and *E. coli*, the MIC values were slightly higher (0.06 and 0.12 mg/mL, respectively). The corresponding values for pure thyme essential oil were 1 mg/mL for *L. monocytogenes* and 2 mg/mL for *S. aureus* and *E. coli*. For Or-CNPs, the lowest MIC values (0.03 mg/mL) were found for both Gram-positive bacteria *L. monocytogenes* and *S. aureus*, while a one-fold higher value (0.06 mg/mL) was found for the Gram-negative bacteria *E. coli*. Moreover, the pure oregano essential oil showed an MIC value of 2 mg/mL for *L. monocytogenes* and 4 mg/mL for *E. coli* and *S. aureus*.

MIC values of CNPs were equal to and slightly lower than Or-CNPs and Th-CNPs, respectively. Examples of chitosan nanoparticles loaded with different essential oils that have greater or even less antimicrobial activity than empty chitosan nanoparticles are reported in the literature [22,45]. In fact, the antibacterial activity of the EO-NPs is due to the contribution of several factors (composition of essential oils, diffusion, surface charge, size, etc.) that do not always act in an additive or synergistic way.

Nevertheless, the results reported in Table 1 showed that for all bacterial strains tested, the antimicrobial activity of EO-CNPs is much greater than free essential oils.

Interestingly, with the exception of Or-CNPs for *S. aureus*, for both nanosystems loaded with essential oils, MBCs values coincided with the MICs values. For Th-CNPs and Or-CNPs, MBC values lower (four- to seven-fold) than pure thyme EO were observed. These results highlighted an excellent bactericidal activity of EO-CNPs on the tested microorganisms, making their use more interesting as natural food preservatives.

In addition, the MBC values of EO-CNPs were also lower than those obtained for the unloaded chitosan nanoparticles. For Th-CNPs and Or-CNPs, MBC values were 0.06 and 0.12 mg/mL for *S. aureus* and *E. coli*, respectively, while for empty CNPs, MBC values ≥ 0.50 mg/mL for the same bacteria were found.

Similarly to what was reported by other authors [6], this significant increase in the bactericidal activity of Th-CNPs and Or-CNPs, with respect to pure EOs and unloaded chitosan NPs, was indicative of a synergistic effect due to the nanometric size (subcellular dimensions) of the chitosan-based nanosystems containing essential oils. Due to interactions between the chitosan cationic group and the anionic components on the cell membrane surface, the chitosan nanoparticles can act as active carriers, facilitating EO diffusion through the bacterial cell membrane.

The mechanism by which essential oils act has been widely studied and the factors involved are numerous. In fact, as previously indicated, EOs have been shown to work with a multi-target mechanism. The composition of the volatile components and their relative concentration strongly influence the biological activity. In particular, carvacrol and thymol, which are the major active compounds present in our samples of thyme essential oil (carvacrol chemotype) and oregano essential oil (thymol chemotype), have been extensively studied for their antimicrobial activity.

The presence of the free OH group is crucial to carry out antimicrobial activity. It was demonstrated that the *p*-cymene compound, is similar in structure to carvacrol and thymol but lacking the hydroxyl group, is less active [46].

The mechanism by which these oxygenated monoterpenes lead to the death of the bacterial cell consists of structural and functional damages to cytoplasmic membrane, including an increase in permeability, leakage of protons and potassium, and depolarization of membrane potential [47,48].

Our data show an increased biocidal activity of Th-CNPs and Or-CNPs compared to pure essential oils and unloaded chitosan nanoparticles, confirming the effectiveness of the nanoencapsulation system as a tool to enhance the antimicrobial activity of essential oils.

## 3. Material and Methods

### 3.1. Materials

Low molecular weight Chitosan (50,000–190,000 Da molecular weight; 75–85% degree of deacetylation) was purchased from Sigma-Aldrich (Milan, Italy); polysorbate 80 (Tween 80) from Merck (Milan, Italy); sodium tripolyphosphate (TPP) from Acros Organics (Geel, Belgium). All chemicals and solvents were of analytic or pharmaceutical grade. EO-CNP suspensions were prepared using LC-MS Grade water (LiChrosolv, Merck, Milan, Italy).

### 3.2. Determination of the Degree of Deacetylation (DD) of Chitosan

The DD of chitosan was determined by ^1^H-NMR spectra recorded on Bruker Avance 400 MHz instruments operating at 400.13 MHz. The sample of chitosan (5 mg) was dissolved in D_2_O/DCl (0.98/0.02 mL). DD was calculated with Equation (1):

(1) $$DD\%=\left[1-\frac{\frac{1}{3}\times HAc}{H2\left(GlcN\right)}\right]\times 100$$

where *HAc* is the integral of the signal corresponding to methyl proton of acetylated units, while *H2*(*GlcN*) is the integral of H_2_ proton of deacetylated units. This formula is recommended for chitosan with *DD* > 48% [49] and when the signal of *H2*(*GlcN*) is sufficiently separated.

### 3.3. Origanum vulgare ssp. hirtum and Thymus capitatus Essential Oils

The essential oils from *Origanum vulgare ssp. hirtum* and *Thymus capitatus,* cultivated plants from Aragona (Sicily), were obtained and characterized as described in our previous published work [29]. Briefly, 100 g of dried aerial part of each sample were subjected to hydrodistillation in a Clevenger-type apparatus (3 h). The obtained oils were dried over anhydrous sodium sulfate, and stored in a sealed vial under N_2_. Gas chromatographic (GC) analyses were performed by a Shimadzu gas chromatograph, Model 17-A, equipped with a flame ionization detector (FID), and an operating software Class VP Chromatography Date System version 4.3 (Shimadzu Italia srl. Milan, Italy). Gas-chromatography-mass spectrometry (GC-MS) analysis was carried out by Shimadzu, GC-MS model GCMS-QP5050A, in the fast mode using the same column and the same operative conditions of GC-FID analysis and software GCMS solution version 1.02 (Shimadzu Italia srl., Milan, Italy). The components were identified by their GC retention index (relative to C_9_–C_20_ *n*-alkanes on the SPB-5 column), computer matching of spectral MS data with those from NIST MS libraries, and the comparison of the fragmentation patterns with those reported in [50]

### 3.4. Preparation of Essential Oil-Loaded Chitosan Nanoparticles (EO-CNPs)

Chitosan (45 mg) were dissolved with 14 mL of acetic acid solution (5.6 mg/mL) by stirring for 30 min (1000 rpm) at 60 °C and sonicating for 60 min by Ultrasonic cleaner 600TH (frequency 45 kHz, power 1200 W; VWR International bvba/sprl, Leuven, Belgium). The obtained chitosan solution (3.2 mg/mL) was filtered through a polypropylene filter (0.6 μm, Millipore, Merck, Milan, Italy) and NaOH 2 N was added to bring the pH to 4.0. 1 mL of aqueous polysorbate 80 (20.5 mg/mL) was poured in 12.5 mL of chitosan solution. The mixture was kept under stirring (1000 rpm) at 60 °C for 30 min and cooled. A total of 1 mL of EO ethanolic solution (35 mg/mL) was dropped in the chitosan mixture under stirring (1200 rpm, for 20 min). Finally, 7.5 mL of TPP aqueous solution (1.87 mg/mL), previously adjusted to pH 4.0 with acetic acid/water (1:1 *v*/*v*), was added dropwise (1 mL/min) under stirring (900 rpm). The stirring was continued for a further 30 min. The empty CNPs were prepared under the same conditions, but in the absence of essential oil.

### 3.5. Characterization of EO-CNPs

#### 3.5.1. Particle Size, Polydispersity Index, and Zeta Potential Measurements

The particle size distribution and the zeta potential (*ζ*) value of nanoparticles were determined at 25 °C by dynamic light scattering (DLS) experiments and electrophoretic mobility, respectively, using a Zetasizer Nano ZS-90, Malvern Instruments, UK. The EO-NCP suspensions were diluted (1:20, *v*/*v*) with pure water. DLS experiments also allowed to calculate the polydispersity index (PDI), which indicates the sample dispersion degree, using the cumulant analysis algorithm. Data analysis was performed using Zetasizer Version 7.02 software.

#### 3.5.2. Viscosity Analysis

The viscosity behavior of the EO-CNP suspensions was observed at 25 °C by a CVO rheometer (Bohlin Instruments, Malvern, UK) equipped with Peltier device for temperature control and with 2°/60 mm diameter steel cone-plate geometry. The results were analyzed by Bohlin R6.51.03 software. The viscosity variation was carried out from 0.1 to 120 s^−1^ shear rate.

#### 3.5.3. Field Emission Scanning Electron Microscopy (FE-SEM)

The morphological characteristics of the nanocapsules were examined through a field-emission scanning electron microscopy (FE-SEM) using a ZEISS SUPRA VP 55 microscope. Samples were prepared by placing a drop of EO-CNP suspensions, diluted with pure water, on a Si substrate. The samples were dried in air and the dried specimens were coated with Au to have conducting samples.

#### 3.5.4. Encapsulation Efficiency (EE) and Loading Capacity (LC) of EO-CNPs

The encapsulation efficiency was calculated using Equation (2):

(2) $$EE\;\left(\%\right)=\frac{\left[EO\;loaded\right]}{{\left[EO\right]}_{tot}}\times 100$$

where [*EO loaded*] = [*EO*]*_tot_* − [*EO*]*_free_* represents the content of essential oil loaded in EO-CNP suspensions and [*EO*]*_tot_* and [*EO*]*_free_* are the total and free contents of essential oil in the EO-CNP suspensions, respectively.

The total content of essential oil in EO-CNP suspensions was determined by UV-Vis spectroscopy (8453 UV-Visible Spectrophotometer, Agilent Technologies, Milan, Italy) over wavelengths ranging from 250 to 450 nm (λ_max_ 274). A total of 100 µL of EO-CNPs was treated with acetonitrile (900 µL), and the resulting mixture was centrifuged (Heraeus Pico 21 centrifuge, Thermo Scientific, Thermo Fisher, Waltham, MA, USA) for 15 min at 3500× *g*. An aliquot of supernatant (400 μL) was diluted with 2 mL of acetonitrile, and the amount of EO was derived by the absorbance at 274 nm using a calibration curve of respective EO (R^2^ = 0.9999), obtained as previously reported (Granata et al., 2018). The total essential oil content of the obtained EO-CNP suspensions was 1.67 mg/mL. The free essential oil was determined by the ultrafiltration/centrifugation technique (Nanosep 30K Omega, Pall Life Science, Milan, Italy; 90 min at 3500× *g*). A total of 500 µL of EO-CNPs was ultrafiltrated, and 200 µL of the filtrate was diluted with 2 mL of acetonitrile. The absorbance at 274 nm of the resulting solution was recorded by a UV-Vis spectrophotometer.

The loading capacity (*LC*) was calculated using Equation (3):

(3) $$LC\;\left(\%\right)=\frac{mass\;of\;loaded\;EO}{mass\;of\;loaded\;nanoparticles\;}\times 100$$

#### 3.5.5. Stability over Time of Essential Oil-Loaded Chitosan Nanoparticles (EO-CNPs)

To evaluate the physicochemical stability of EO-CNPs over time, the particle size distribution (PSD), zeta potential, and loaded amount retention (%) of EOs in CNPs were checked every week for a period of 30 days, on samples kept at different temperatures (4 °C and 40 °C).

In particular, the EO retention (%) was determined according to Equation (4):

(4) $$EO\;retention\;\left(\%\right)=\frac{{\left[EO\;loaded\right]}_{t}}{{\left[EO\;loaded\right]}_{0}}\times 100$$

where [*EO loaded*]*_t_* is the EO loaded concentration at the storage time *t* and [*EO loaded*]*_0_* is the loaded concentration of freshly prepared nanosuspensions.

#### 3.5.6. Statistical Analysis

Each experiment was replicated at least twice and measurements were performed in triplicate. All data are presented as mean ± standard deviation (SD).

Analysis of variance (ANOVA) followed by Tukey’s test at a significance level of 0.05 were performed for the statistical evaluation of the experimental stability data.

### 3.6. Microbiological Studies

#### 3.6.1. Bacterial Strains and Growth Conditions

All the tested strains, i.e., *Staphylococcus aureus* ATCC 29213, *Escherichia coli* ATCC 25922, and *Lysteria monocytogenes* ATCC 19118, were purchased from the American type Culture Collection (Manassas, VA, USA). *S. aureus* ATCC 29213 colonies were cultured on MSA (Oxoid, Basingstoke, UK) plates, *E. coli* ATCC 25922 and *L. monocytogenes* ATCC 19118 were cultured on BHI agar (Oxoid, Basingstoke, UK) plates. All the strains were incubated at 37 °C for 18 h.

#### 3.6.2. Minimum Inhibitory Concentration (MIC) and Minimum Bactericidal Concentration (MBC)

The minimum inhibitory concentration of the Th-CNPs and Or-CNPs and empty CNPs was calculated by microtiter plate assays according to the standard method [51], with minor modifications.

EO-CNP aqueous suspensions (1.67 mg/mL of essential oil) and empty CNPs were diluted in Mueller Hinton broth (MHB) (Oxoid, Basingstoke, UK) or BHI broth (BHIB) (Oxoid, Basingstoke, UK) for *L. monocytogenes* to a 1 mg/mL concentration and serial two-fold dilutions were performed in 96-well microplates containing sterile MHB or BHIB. A few freshly streaked (less than 24 h) bacterial colonies were resuspended in sterile distillate water, and then the suspensions were opportunely diluted in the appropriate media to reach a turbidity equal to 10^6^ CFU/mL. This was next transferred to the 96-well microplate, reaching a bacterial concentration of 10^5^ CFU/mL in each well. A total of 100 μL of the 106 CFU/mL suspension, appropriately diluted, was spotted on MH or BHI plates for the MBC determination. Finally, the microplates were incubated for 18 h at 37 °C. Empty CNPs were used as chitosan antimicrobial activity controls and wells with sterile MHB or BHI as sterility controls. MIC and MBC values were determined as previously published [29] and expressed as mg/mL. All assays were performed in triplicate with independently grown cultures. The results showed that such a high reproducibility such that when calculating a mean was unnecessary.

## 4. Conclusions

The goal of this study is to obtain efficient natural systems with the antibacterial activity for health and food safety applications. By ionic gelation technique, we prepared chitosan nanoparticles loaded with *Thymus capitatus* (carvacrol chemotype) and *Origanum vulgare* (thymol chemotype), in the presence of non-toxic crosslinking/gelling agent (TPP). The Th-CNP and Or-CNP nanosytems showed good encapsulation efficiency with values of 80 ± 2% and 83 ± 3%, and a loading capacity of 26 ± 1% and 27 ± 1%, respectively. The prepared nanoparticles have a spherical shape and a bimodal particle size distribution. The zeta potential, which was greater than 40 mV, indicated the good stability and homogeneity of both nanosystems. The EO-NCPs were able to act as reservoir systems for long-term protection of essential oils, and Th-NCPs are more efficient than Or-CNPs. Both the preparations showed an increased biocidal activity against foodborne pathogens (*E. coli*, *S. aureus*, and *L. monocytogenes*), compared to pure essential oils and empty chitosan nanoparticles.

These results highlight the effectiveness of nanoencapsulation of essential oils in biodegradable and biocompatible natural polymer such as chitosan. Therefore, the prepared chitosan nanoparticles loaded with essential oils of thyme or oregano could be a viable alternative to synthetic preservatives when very close control of severe pathogen contamination is required.

## Figures and Tables

**Table 1:** MIC ^a^ and MBC ^a^ of essential oils chitosan nanoparticle suspensions (EO-CNPs) and empty chitosan nanoparticle suspensions (CNPs) against different microbial strains.

	*S. aureus* ATCC 29213		*E. coli* ATCC 25922		*L. monocytogenes* ATCC 19118	
	MIC	MBC	MIC	MBC	MIC	MBC
Th-CNPs	0.06	0.06	0.12	0.12	0.03	0.03
Th-EO	2	2	2	2	1	2
Or-CNPs	0.03	0.06	0.06	0.06	0.03	0.03
Or-EO	4	4	4	4	2	4
CNPs	0.03	>0.50	0.06	0.50	0.03	0.06

^a^ MIC and ^a^ MBC are expressed in mg/mL.

## Data Availability

The data presented in this study are available in this article.

## Supplementary Materials

The following are available online. Figure S1: ^1^H NMR spectrum of chitosan in DCl/D_2_O at 343 K. HAc is the signal corresponding to methyl proton of acetylated units; H2(GlcN) is the H2 proton of deacetylated units, Table S1: GC-MS analysis of thyme and oregano essential oil, Table S2. Stability over time of thyme essential oil-loaded chitosan nanoparticles (Th-CNPs) at 4 °C, Table S3. Stability over time of oregano essential oil-loaded chitosan nanoparticles (Or-CNPs) at 4 °C, Table S4. Stability over time of thyme essential oil-loaded chitosan nanoparticles (Th-CNPs) at 40 °C, Table S5. Stability over time of oregano essential oil-loaded chitosan nanoparticles (Or-CNPs) at 40 °C, Figure S2. Plot of the viscosity versus shear rate for the Or-CNP suspension.
