# Supplementary material for: Oregano and Thyme Essential Oils Encapsulated in Chitosan Nanoparticles as Effective Antimicrobial Agents against Foodborne Pathogens
Source: Molecules. 2021 Jul 2;26(13):4055. doi: 10.3390/molecules26134055 (PMC8271874; doi:10.3390/molecules26134055)
Supplement: Supplementary file 1 [file molecules-26-04055-s001.zip › molecules-1281274-supplementary.pdf]

Chitosan nanoparticles as effective reservoir of thyme and oregano essential oils with antibacterial activity against foodborne pathogens

Giuseppe Granata, Stefano Stracquadanio, Marco Leonardi, Edoardo Napoli, Graziella Malandrino, Viviana Cafiso, Stefania Stefani, Corrada Geraci*

Supplementary Data

Figure and Table Captions

Figure S1. ^{1}H NMR spectrum of chitosan in DCl/D_2O at 343 K. HAc is the signal corresponding to methyl proton of acetylated units; H2(GlcN) is the H2 proton of deacetylated units.

Table S1. GC-Ms analysis of thyme and oregano essential oil

Table S2. Stability over time of thyme essential oil-loaded chitosan nanoparticles (Th-CNPs) at 4°C.

Table S3. Stability over time of oregano essential oil-loaded chitosan nanoparticles (Or-CNPs) at 4°C.

Table S4. Stability over time of thyme essential oil-loaded chitosan nanoparticles (Th-CNPs) at 40°C.

Table S5. Stability over time of oregano essential oil-loaded chitosan nanoparticles (Or-CNPs) at 40°C.

Figure S2. Plot of the viscosity versus shear rate for the Or-CNP suspension.

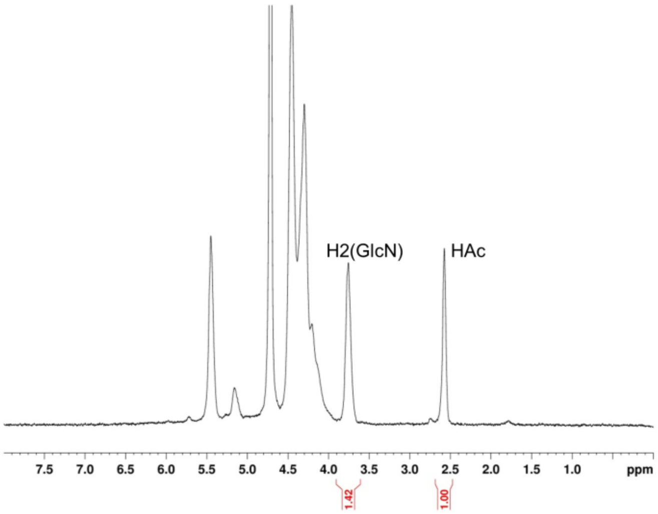

Figure S1. ^{1}H NMR spectrum of chitosan in DCl/D_2O at 343 K. HAc is the signal corresponding to methyl proton of acetylated units; H2(GlcN) is the H2 proton signal of deacetylated units.

Table S2. GC-MS analysis of oregano and thyme essential oil[a]

**Compound[b]**	**Oregano (Or-EO) %[c]**	**Thyme (Th-EO) %[c]**
α-Thujene	1.26 (±0.005)	0.67 (±0.003)
Myrcene	2.28 (±0.008)	1.71 (±0.005)
α-Terpinene	2.48 (±0.008)	1.18 (±0.003)
p-cymene	14.49 (± 0.038)	6.88 (±0.014)
cis-Ocimene	1.75 (±0.004)	-
γ-Terpinene	15.35 (±0.031)	2.50 (±0.006)
Linalool	0.63 (±0.001)	1.17 (0.006)
Thymol methyl ether	1.99 (±0.002)	-
Carvacrol methyl ether	3.64 (±0.003)	-
Thymol	43.93 (±0.106)	0.31 (±0.025)
Carvacrol	0.51 (±0.002)	73.03 (±0.055)
β-Caryophyllene	1.40 (±0.001)	5.45 (±0.005)
β-Bisabolene	1.69 (±0.003)	0.49 (±0.001)
δ-Cadinene	1.04 (±0.003)	0.07 (±0.001)
**Monoterpene hydrocarbons**	**40.31 (±0.095)**	**14.68 (±0.032)**
**Oxygenated monoterpenes**	**52.12 (±0.111)**	**76.61 (±0.052)**
**Sesquiterpenes**	**7.36 (±0.188)**	**7.15 (±0.013)**
**Others**	**0.02 (±0.001)**	**0.95 (±0.001)**

[a]Data previously reported (see Granata et al. 2018a)
[b]Identified compounds with relative percentages <1.0 % in both samples were not listed.
[c]Relative peak area percent represents averages of 3 determinations.

Table S2. Stability over time of thyme essential oil-loaded chitosan nanoparticles (Th-CNPs) at 4°C.

Th-CNPs	Storage time (days)				
	0	7	15	21	30
PSD peak 1 (nm)	86 (22)	59 (11)	55 (10)	88 (22)	73 (15)
PSD peak 2 (nm)	449 (205)	459 (185)	448 (198)	463 (205)	450 (214)
ζ (mV)	+44 ± 2[a]	+36 ± 1[b]	+45 ± 1[a]	+42 ± 2[a,c]	+39 ± 1[b,c]
Th loaded amount (mg/mL)	1.33 ± 0.03[a]	1.33 ± 0.06[a]	1.30 ± 0.08[a]	1.26 ± 0.03[a]	1.21 ± 0.05[a]

PSD = Particle Size (hydrodynamic diameter) Distribution, in brackets the peak width value.
Values in the same line with the same superscripts are not significantly different ($p > 0.05$).

Table S3. Stability over time of oregano essential oil-loaded chitosan nanoparticles (Or-CNPs) at 4°C.

Or-CNPs	Storage time (days)				
	0	7	15	21	30
PSD peak 1 (nm)	62 (19)	82 (24)	58 (14)	53 (19)	96 (27)
PSD peak 2 (nm)	407 (191)	473 (202)	468 (224)	473 (263)	434 (185)
ζ (mV)	+46 ± 2[a]	+48 ± 1[a,b]	+42 ± 2[a,c]	+45 ± 2[a,b,c]	+41 ± 1[c]
Or loaded amount (mg/mL)	1.38 ± 0.05[a]	1.38 ± 0.03[a]	1.26 ± 0.05[a]	1.10 ± 0.04[b]	0.98 ± 0.09[b]

PSD = Particle Size (hydrodynamic diameter) Distribution, in brackets the peak width value.
Values in the same line with the same superscripts are not significantly different ($p > 0.05$).

Table S4. Stability over time of thyme essential oil-loaded chitosan nanoparticles (Th-CNPs) at 40°C.

Th-CNPs	Storage time (days)				
	0	7	15	21	30
PSD peak 1 (nm)	86 (22)	66 (16)	68 (16)	84 (21)	60 (14)
PSD peak 2 (nm)	449 (205)	442 (248)	462 (206)	428 (174)	474 (233)
ζ (mV)	+44 ± 2[a]	+45 ± 1[a,b]	+45 ± 1[a,b]	+41 ± 1[a,c]	+40 ± 1[c]
Th loaded amount (mg/mL)	1.33 ± 0.03[a]	1.30 ± 0.04[a]	1.28 ± 0.05[a]	1.26 ± 0.06[a,b]	1.11 ± 0.09[b]

PSD = Particle Size (hydrodynamic diameter) Distribution, in brackets the peak width value.
Values in the same line with the same superscripts are not significantly different ($p > 0.05$).

Table S5. Stability over time of oregano essential oil-loaded chitosan nanoparticles (Or-CNPs) at 40°C.

Or-CNPs	Storage time (days)				
	0	7	15	21	30
PSD peak 1 (nm)	62 (19)	45 (12)	78 (19)	52 (13)	90 (21)
PSD peak 2 (nm)	407 (191)	394 (229)	494 (267)	295 (147)	305 (136)
ζ (mV)	$+46 \pm 2^{a}$	$+46 \pm 1^{a}$	$+42 \pm 1^{b}$	$+41 \pm 1^{b}$	$+42 \pm 1^{b}$
Or loaded amount (mg/mL)	1.38 ± 0.05^{a}	0.72 ± 0.04^{b}	0.70 ± 0.09^{b}	0.59 ± 0.04^{b}	0.43 ± 0.02^{c}

PSD = Particle Size (hydrodynamic diameter) Distribution, in brackets the peak width value.
Values in the same line with the same superscripts are not significantly different ($p > 0.05$).

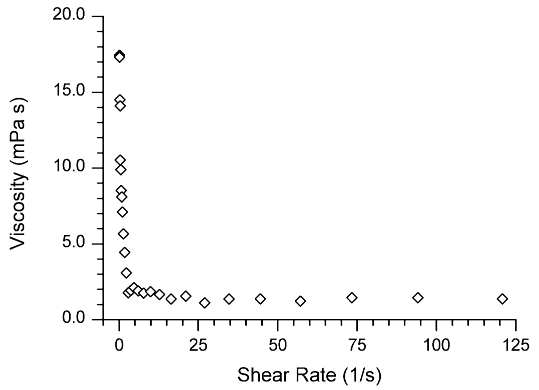

Figure S2. Plot of the viscosity versus shear rate for the Or-CNP suspension.
